# Generative AI–Assisted Microlearning for Erectile Dysfunction Myth Reduction: Single-Center Pre–Post Quasi-Experimental Study

**DOI:** 10.2196/88818

**Published:** 2026-05-14

**Authors:** Ali Can Albaz, Oğuzcan Erbatu, Okan Yiğit, Oktay Üçer, Gökhan Temeltaş, Talha Müezzinoğlu

**Affiliations:** 1 Department of Urology, Faculty of Medicine, Manisa Celal Bayar University, No:189 Mimarsinan Street, Uncubozköy, Yunusemre, Manisa 45030, Türkiye, Manisa, Manisa, 45030, Turkey, 90 5059129003

**Keywords:** erectile dysfunction, sexual-health literacy, generative artificial intelligence, large language model, microlearning, digital health education, misinformation, urology, pre–post study

## Abstract

**Background:**

Erectile dysfunction (ED) is strongly influenced by persistent misconceptions that delay help-seeking and limit engagement with effective care. Patient-centered digital strategies, including generative artificial intelligence (AI) microlearning, may improve sexual-health knowledge; however, real-world evidence in urological practice remains sparse.

**Objective:**

This study aimed to evaluate whether a clinician-supervised generative AI microlearning video improves ED-related knowledge in adult men attending routine outpatient care.

**Methods:**

This single-center pre–post quasi-experimental study included 200 adult men in a university urology clinic. Participants completed an 8-item ED myth questionnaire immediately before and after watching a 3-minute educational video. The narration script was drafted using a large language model (ChatGPT) and iteratively reviewed by urologists for accuracy and cultural appropriateness. The primary outcome was the within-participant change in total correct responses (0‐8). Subgroup analyses assessed effects across age (<40 years vs ≥40 years), education level, and self-reported ED. Paired analyses and multivariable logistic regression were used (α=.05).

**Results:**

All participants completed the intervention (mean age 44.0, SD 11.6 years). Total mean correct responses increased from 3.77 to 6.56 (mean difference 2.79; *P*<.001), indicating a large effect (Cohen *d*=1.52). Knowledge gains were consistent across subgroups, with greater improvements among those with lower education. Self-reported ED was independently associated with lower odds of achieving ≥2-point improvement (odds ratio 0.46, 95% CI 0.26‐0.81; *P*=.01). No adverse events or technical difficulties occurred.

**Conclusions:**

A brief clinician-supervised generative AI microlearning video was associated with substantial short-term improvements in ED myth–related knowledge in routine outpatient care. AI-assisted microlearning may represent a scalable adjunct to patient education during urological consultations. Future studies should evaluate long-term retention and behavioral outcomes.

## Introduction

Erectile dysfunction (ED) is a multidimensional health problem with psychological, physiological, and relational consequences, adversely affecting sexual well-being and quality of life in men. Epidemiological studies have consistently shown that ED is highly prevalent and strongly age-related. In the Massachusetts Male Aging Study, approximately half of men aged 40 to 70 years reported some degree of ED [1,2]. Likewise, population-based studies in Brazil and Europe have estimated prevalence rates between 30% and 50% [3,4]. Beyond impairing sexual function, ED significantly disrupts self-esteem, intimate relationships, and psychosocial health [5,6].

Culturally reinforced myths about ED remain widespread. Common misconceptions include viewing ED as an inevitable consequence of aging, believing it has no effective treatments, assuming that medications are unsafe, or attributing ED solely to psychological causes [7,8]. Such beliefs discourage men from seeking medical attention, reduce treatment adherence, and undermine therapeutic trust. In cultures where sexuality is stigmatized, embarrassment and social taboos further delay clinical consultation [9]. Thus, ED-related myths not only perpetuate unhealthy beliefs but also contribute to avoidable diagnostic delays and preventable declines in sexual-health outcomes. The literature consistently demonstrates that insufficient knowledge about ED negatively affects help-seeking and lowers sexual-health literacy [10,11].

Digital health communication strategies have emerged as scalable solutions for health education and myth reduction. Visually enriched microlearning videos and mobile health apps have demonstrated improvements in men’s sexual-health knowledge [12]. Such tools are inexpensive, accessible, and capable of reaching large populations—making them valuable for public health implementation [13,14]. However, the impact of these interventions is contingent upon the accuracy and credibility of their content.

Advances in artificial intelligence (AI) have introduced new opportunities to optimize digital health education. Large language model (LLM) systems such as ChatGPT (OpenAI) can simplify complex medical concepts, enhance comprehension, and potentially counteract misinformation [15]. AI-generated educational resources may be particularly beneficial for individuals with limited health literacy [16-18]. Emerging evidence from medical education and patient communication research suggests that generative AI tools can improve accessibility, relevance, and user satisfaction [19,20].

Despite the substantial burden of misconceptions surrounding ED, the educational impact of AI-assisted microlearning interventions in real-world clinical settings remains insufficiently examined. Therefore, the aim of this study was to evaluate whether a brief clinician-supervised AI-assisted educational video could improve knowledge related to common ED myths among adult men attending an outpatient urology clinic. By addressing this gap, the study explores the potential of generative AI as a scalable adjunct to sexual-health education.

## Methods

### Ethical Considerations

The study protocol was approved by the Institutional Clinical Research Ethics Committee of Manisa Celal Bayar University, Faculty of Medicine (20.478.486/3376, dated August 27, 2025), and conducted in accordance with the ethical principles of the Declaration of Helsinki. Written informed consent was obtained from all participants prior to enrollment. All data were collected anonymously, no personally identifiable information was recorded, and all data were handled in accordance with institutional and ethical standards. Participation was voluntary, and no financial compensation was provided.

### Study Design

This single-center pre–post quasi-experimental study was conducted at the outpatient urology clinic of Manisa Celal Bayar University Faculty of Medicine (Manisa, Türkiye) between August 30 and September 30, 2025. The study was not prospectively registered because it did not meet the criteria for a clinical trial involving therapeutic intervention or alteration of patient management.

### Participants

Adult men aged 18 years or older attending the outpatient urology clinic for routine evaluation, irrespective of visit reason, were invited to participate. Eligible participants were approached consecutively during routine outpatient clinic hours by trained research staff using a standardized recruitment script. Eligibility criteria included Turkish literacy and completion of both preintervention and postintervention questionnaires following full exposure to the educational video. Exclusion criteria were aged younger than 18 years, incomplete questionnaires, or cognitive or communication barriers preventing informed participation. A target sample size of 200 participants was determined on feasibility grounds and achieved within the study period.

### Intervention

The intervention consisted of a 3-minute educational microlearning video designed to address 8 commonly encountered myths related to ED. The initial narration script was drafted using a LLM (ChatGPT) through a structured prompting approach. The prompts explicitly instructed the model to generate medically accurate explanations correcting predefined ED misconceptions using clear, patient-oriented, nontechnical language. Additionally, the prompts incorporated predefined ED misconceptions alongside evidence-based corrective explanations aligned with contemporary clinical knowledge. The prompts were predefined and standardized to ensure consistency of AI-generated content.

The educational video addressed 8 common misconceptions regarding ED. These included beliefs that ED is an inevitable consequence of aging, that effective treatments are unavailable, that pharmacological therapies are unsafe, and that ED is exclusively psychological in origin. Each misconception was briefly presented and immediately followed by a concise evidence-based explanation correcting the myth. The educational structure followed a myth-correction format combining short narrative segments with simple visual elements designed to improve comprehension.

The AI-generated script underwent clinician review by 2 board-certified urologists. The review process evaluated factual accuracy, consistency with contemporary clinical knowledge, linguistic clarity, and cultural appropriateness. Revisions were made when necessary, and any discrepancies between the AI-generated content and clinician judgment were resolved through consensus prior to final video production.

During this review process, the clinicians compared the AI-generated explanations with current evidence-based urological knowledge and relevant guideline-based recommendations. Minor revisions were made to improve medical accuracy, terminology, and clarity for a lay audience. These revisions primarily involved correcting oversimplified statements and ensuring that treatment-related information reflected established clinical practice.

The final video incorporated narrated explanations supported by brief animated and static visual elements with captions. Participants viewed the video individually on a tablet or computer in a quiet consultation room under staff supervision to ensure standardized exposure and minimize distractions.

### Outcome Measures

ED myth–related knowledge was evaluated using an 8-item questionnaire developed through literature review and expert consultation to optimize clarity and content validity. Each item was presented as a statement with response options “true,” “false,” or “don’t know.” Items representing misconceptions (myths) were reverse-coded such that medically correct responses were scored as 1 and incorrect or “don’t know” responses as 0. Total scores ranged from 0 to 8.

The questionnaire was administered immediately before (pretest) and after (posttest) the intervention. The immediate postintervention assessment was intentionally selected to evaluate short-term knowledge acquisition rather than long-term retention.

The primary outcome was the within-participant change in total correct responses. Secondary analyses explored differences across age groups (<40 years vs ≥40 years), educational attainment levels (primary or middle school, high school, university, and postgraduate), and the presence of self-reported erectile difficulties within the past year.

### Data Management

All questionnaires were anonymized using coded identifiers. Data integrity and accuracy were supported by predefined range and logic checks. Duplicate verification was conducted for a random 10% subset of records before statistical analysis.

### Statistical Analysis

Statistical analyses were performed using SPSS (version 29.0; IBM Corp). Continuous variables were summarized as mean (SD) or median (IQR) following assessment of distribution using the Shapiro-Wilk test. Pre–post changes in total scores were evaluated using paired 2-tailed *t* tests or Wilcoxon signed-rank tests as appropriate. Effect sizes were reported as Cohen *d* or r with 95% CIs. Item-level comparisons were conducted using McNemar tests with Holm multiplicity adjustment. Between-group comparisons used independent *t* tests or one-way ANOVA for normally distributed variables and appropriate nonparametric alternatives otherwise. Multivariable logistic regression analysis was used to identify independent predictors of improvement, with age, education level, and self-reported ED status entered as covariates. A post hoc power calculation confirmed that the achieved sample size provided >90% power to detect a paired change of ≥0.4 SDs.

This study is reported in accordance with the TREND (Transparent Reporting of Evaluations with Nonrandomized Designs) statement for nonrandomized evaluations.

### Patient and Public Involvement

Patients and the public were not involved in the design of the study or the selection of the outcomes assessed. However, the clinic staff reviewed the questionnaire wording and the video narration to enhance comprehensibility and cultural appropriateness.

### Use of Generative AI

The narration script of the educational video was initially generated using ChatGPT. The AI-generated content was critically reviewed, revised, and approved by board-certified urologists to ensure factual accuracy, clinical appropriateness, clarity, and cultural relevance. No generative AI tools were used for data analysis, statistical evaluation, or interpretation of the study findings.

## Results

### Participant Characteristics

A total of 200 men were enrolled between August 30 and September 30, 2025. The mean age was 44.0 (SD 11.6) years (range 18‐75), and 118 (59%) were aged ≥40 years. Self-reported ED during the previous year was noted in 97 (48.5%) participants. Regarding education levels, 66 (33%) participants had completed primary or middle school, 74 (37%) had completed high school, 48 (24%) had finished university, and 12 (6%) held a postgraduate degree.

Table 1 summarizes baseline characteristics.

Paired sample *t* test was used for normally distributed variables and Wilcoxon signed-rank test was used for nonparametric distributions. Proportional improvements were evaluated using McNemar analysis (Table 2).

**Table 1.** Baseline characteristics of the study participants (N=200)^a^.

Characteristics	Participants
Age (y), mean (SD; range)	44.0 (11.6; 18-75)
Age group, n (%)	
<40 y	82 (41)
≥40 y	118 (59)
Self-reported erectile difficulties in the past year, n (%)	
Yes	97 (48.5)
No	103 (51.5)
Educational level, n (%)	
Primary or middle school	66 (33)
High school	74 (37)
University	48 (24)
Postgraduate	12 (6)

a Data are presented as mean (SD) for continuous variables and as number (percentage) for categorical variables.

**Table 2.** Preintervention and postintervention erectile dysfunction (ED) myth–related knowledge scores.

Measurement	Before intervention	After intervention	Mean change (SD)	*P* value^a^
Total ED myth–related knowledge score, mean (SD)	3.77 (1.65)	6.56 (1.44)	2.79 (1.85)	<.001^b^
Correct responses (%), mean (SD)	25.1 (10.9)	43.7 (9.6)	18.6 (12.3)	<.001^b^
Score^c^, median (IQR)	4 (3-5)	7 (6-8)	—^d^	<.001^e^
Participants improving ≥2 points (n=200), n (%)	—	84 (42)	—	.004^f^

a *P* values were derived from paired comparisons; Holm correction was applied for multiple testing.

b Paired *t* test.

c Score refers to the total number of correct responses on the erectile dysfunction myth–related knowledge questionnaire, with higher scores indicating greater knowledge.

d Not applicable.

e Wilcoxon signed-rank test.

f McNemar test.

### Overall Change in Knowledge

Following the educational intervention, participants demonstrated a significant improvement in their knowledge scores about ED-related misconceptions. The mean score increased from 3.77 (SD 1.65) at pretest to 6.56 (SD 1.44) at posttest (mean change 2.79, SD 1.85; *P*<.001). The within-subject effect size was large (Cohen *d*=1.52; 95% CI 1.32‐1.72). These results are presented in Table 2.

### Subgroup Results by Age and Education

Improvements were observed across all age and education strata, as shown in Table 3.

**Table 3.** Pre–post changes in knowledge by age and educational level^a^.

Subgroup	Pretest, mean (SD)	Posttest, mean (SD)	Mean change (SD)	*P* value
Primary or middle school (n=66)	3.10 (1.78)	6.10 (1.53)	3.00 (2.10)	<.001
High school (n=74)	3.55 (1.65)	7.00 (1.42)	3.45 (1.87)	<.001
University (n=48)	4.60 (1.61)	6.55 (1.38)	1.95 (1.72)	<.001
Postgraduate (n=12)	5.50 (1.50)	6.50 (1.30)	1.00 (1.41)	.02
<40 y (n=82)	3.90 (1.70)	6.90 (1.40)	3.00 (1.80)	<.001
≥40 y (n=118)	3.20 (1.60)	6.00 (1.50)	2.80 (1.90)	<.001

a Participants with lower educational attainment showed lower baseline scores but greater absolute gains.

### Differences According to Self-Reported ED

Knowledge improvements were significant in both ED-positive and ED-negative groups, as shown in Table 4.

**Table 4.** Pre–post changes by erectile dysfunction (ED) status^a^.

ED status	Pretest, mean (SD)	Posttest, mean (SD)	Mean change (SD)	*P* value
ED positive (n=97)	3.20 (1.60)	6.00 (1.50)	2.80 (1.90)	<.001
ED negative (n=103)	3.90 (1.70)	6.90 (1.40)	3.00 (1.80)	<.001

a Effect sizes remained large in both subgroups.

### Predictors of Improvement

In multivariable logistic regression, self-reported ED was independently associated with lower odds of ≥2-point improvement (odds ratio 0.46, 95% CI 0.26-0.81; *P*=.01), as shown in Table 5. Neither age (*P*=.28) nor educational level (*P*=.37) were significant predictors.

**Table 5.** Predictors of improvement in multivariable analysis.

Variables	Odds ratio (95% CI)		*P* value
Age ≥40 y	1.40 (0.76‐2.59)		.28
Education level (ordinal increase)	0.87 (0.64‐1.18)		.37
Self-reported erectile dysfunction (yes vs no)	0.46 (0.26‐0.81)		.01

### Summary of Principal Findings

All participants demonstrated significant reductions in ED-related misconceptions following the brief AI-generated educational video, regardless of age, educational level, or presence of ED.

## Discussion

### Principal Findings

This study demonstrates that a brief AI-generated educational video can meaningfully reduce common misconceptions about ED in diverse male outpatients. The intervention produced large and statistically significant knowledge gains in every subgroup, with the most pronounced absolute improvements among participants with lower educational attainment. These findings confirm that concise, visually supported, and expert-supervised AI-generated content can effectively correct persistent sexual-health misinformation, a domain where stigma and cultural taboos often limit traditional educational outreach.

### Interpretation of Findings

ED remains a biopsychosocial condition deeply intertwined with masculine identity, relationship dynamics, and sociocultural context [21]. Misbeliefs such as “ED is an unavoidable consequence of aging,” “there is no effective treatment,” or “medications are unsafe” have been widely documented as deterrents to clinical consultation [22,23]. These data show that such entrenched ideas can be corrected through a 3-minute, evidence-based video combining verbal and visual explanations—a format consistent with established debunking principles that emphasize factual alternatives and avoid myth repetition [22,23].

The magnitude of improvement observed in this study aligns with prior meta-analyses reporting that video-based health communication enhances comprehension, recall, and behavioral intent compared with static or text-only materials [24,25]. According to multimedia learning theory, dual-channel processing of verbal and visual information reduces cognitive load and facilitates durable understanding. This intervention capitalized on this mechanism by combining AI-generated narrative clarity with succinct animations and culturally sensitive wording, which likely contributed to the robust pre–post effect sizes.

From a public health perspective, the greater benefit among men with lower education reflects the health literacy gradient described in prior systematic reviews [14,15]. Health literacy is a crucial determinant of individuals’ ability to obtain, process, and act upon medical information; interventions that simplify complex content yield the largest gains among those starting from the lowest literacy levels. The AI-based video functioned as a scalable equalizer—translating specialist concepts into accessible language without sacrificing accuracy. In contexts where clinical time is limited, these microlearning tools can complement consultation, enhancing patient understanding before physician interaction.

Interestingly, participants who self-reported ED achieved significant absolute gains but showed a lower adjusted probability of improvement in multivariable analysis. This paradox may stem from anchoring effects, where preexisting illness experience, embarrassment, or prior misconceptions reduce openness to new information [26,27]. Nevertheless, the measurable knowledge increase in this group underscores that even emotionally charged topics can be addressed effectively when conveyed through empathic, stigma-neutral communication. Integrating brief AI-generated videos before counseling sessions could therefore enhance patient readiness and engagement during discussion of treatment options.

Beyond its educational utility, this study highlights the transformative yet bounded role of generative AI in patient communication. LLM-based systems are capable of producing coherent, high-readability medical explanations at scale; however, their unsupervised outputs may contain factual inaccuracies or contextual distortions [28,29]. By pairing algorithmic drafting with urologist review and citation to peer-reviewed sources, the current design mitigated such risks. This hybrid model—AI generation under expert oversight—illustrates a pragmatic framework for safely incorporating generative AI into urological practice and patient education.

The observed educational gains are further supported by prior research demonstrating that brief digital interventions can improve health knowledge and self-efficacy, particularly when designed for high readability and low cognitive burden [30-32]. In sexual-health contexts, where embarrassment and stigma frequently constrain direct clinician-patient dialogue, asynchronous video-based microlearning tools may provide a psychologically safer entry point for information exposure. This mechanism may partly explain the substantial within-participant improvements observed in this study.

Age-based analyses revealed a similar magnitude of improvement across younger and older participants, suggesting that chronological age per se is not a limiting factor for digital learning. Rather, comprehension appears to depend more on cognitive load, clarity of message, and design coherence, consistent with prior evidence indicating that well-structured digital materials can benefit older adults as effectively as younger ones [31]. The uniform effect across age strata further supports the adaptability of AI-mediated educational interventions across demographic boundaries.

This study has several methodological strengths, including an adequate sample size, a clearly prespecified primary outcome (change in total correct responses), stratified subgroup analyses by education, age, and ED status, and the use of multivariable logistic regression to explore independent predictors of improvement. The consistently large effect sizes (Cohen *d*>1.0) further support the robustness of the observed within-participant learning gains.

Nevertheless, several limitations should be considered when interpreting these findings. The pre–post design lacked a randomized control arm, introducing potential testing, recall, or expectancy effects. Only short-term knowledge acquisition was assessed immediately after the intervention; longer-term durability and behavioral translation (eg, clinical consultation, treatment initiation, or partner communication) remain unknown. Immediate postintervention testing primarily reflects short-term recall rather than consolidated long-term knowledge. Educational research indicates that improvements observed immediately after an instructional exposure may partly reflect transient memory effects rather than durable knowledge acquisition. Therefore, the magnitude of improvement reported in this study should be interpreted as short-term knowledge gain following exposure to the educational video. Future studies incorporating delayed follow-up assessments would help determine whether these improvements persist over time and translate into sustained sexual-health literacy. The sample was drawn from a single center in Türkiye, possibly limiting cross-cultural generalizability. Socioeconomic status, digital literacy, and comorbid conditions were not included in the regression model and may have influenced responsiveness to the intervention. Moreover, the intervention used a single 3-minute format; alternative narrative framings, interactivity, and dissemination channels (waiting-room screens, mobile apps, or patient portals) warrant future exploration. Although interaction effects between ED status, age, and education were not modeled, the multivariable regression analysis partially mitigates confounding by simultaneously adjusting for these covariates.

Future multicenter randomized controlled trials are warranted to compare AI-generated versus clinician-written or conventional educational materials, assess retention at delayed follow-ups, and measure behavioral and psychological end points. Cost-effectiveness and equity analyses are also needed to guide implementation in resource-limited settings.

Taken together, these findings indicate that a brief, clinician-supervised educational video generated using a generative AI platform (ChatGPT-based) can deliver substantive and equitable gains in ED-related knowledge across routine outpatient settings, with the largest absolute benefits observed among men with lower educational attainment. Within the limits of a single-center pre–post design, these results support pragmatic use of AI-assisted microlearning as a scalable adjunct to counseling in urology. Rigorous multicenter randomized studies with longer follow-up are now needed to determine durability, generalizability, and real-world implementation value.

### Conclusions

In this single-center pre–post study, a 3-minute clinician-supervised educational video generated using a generative AI platform was associated with a significant reduction in belief in common ED myths among adult men. Knowledge gains were observed across demographic subgroups, with the largest absolute improvements among participants with lower educational attainment. These findings suggest that brief, expert-supervised AI-assisted microlearning may serve as a feasible adjunct to patient education in outpatient urology settings. Further multicenter randomized controlled studies with longer follow-up are warranted to assess durability of knowledge retention, behavioral outcomes, and cost-effectiveness.

## Abbreviations

AI: artificial intelligence
ED: erectile dysfunction
LLM: large language model
TREND: Transparent Reporting of Evaluations with Nonrandomized Designs
